# Single-Energy Metal Artifact Reduction (SEMAR) in Ultra-High-Resolution CT Angiography of Patients with Intracranial Implants

**DOI:** 10.3390/diagnostics13040620

**Published:** 2023-02-08

**Authors:** Abdullah Jabas, Mario Alberto Abello Mercado, Sebastian Altmann, Florian Ringel, Christian Booz, Andrea Kronfeld, Antoine P. Sanner, Marc A. Brockmann, Ahmed E. Othman

**Affiliations:** 1 Department of Neuroradiology, University Medical Center Mainz, Langenbeckstraße 1, 55131 Mainz, Germany; 2 Department of Neurosurgery, University Medical Center Mainz, Langenbeckstraße 1, 55131 Mainz, Germany; 3 Department of Diagnostic and Interventional Radiology, University Hospital Frankfurt, 60590 Frankfurt, Germany; 4 Department of Computer Science, Technical University Darmstad, Fraunhofer IGD, Fraunhoferstraße 5, 64283 Darmstadt, Germany

**Keywords:** metal artifact reduction, artifacts, computed tomography angiography, ultra-high-resolution computed tomography, intracranial aneurysm

## Abstract

Purpose: To evaluate the effects of single-energy metal artifact reduction (SEMAR) on image quality of ultra-high-resolution CT-angiography (UHR-CTA) with intracranial implants after aneurysm treatment. Methods: Image quality of standard and SEMAR-reconstructed UHR-CT-angiography images of 54 patients who underwent coiling or clipping was retrospectively evaluated. Image noise (i.e., index for metal-artifact strength) was analyzed in close proximity to and more distally from the metal implant. Frequencies and intensities of metal artifacts were additionally measured and intensity-differences between both reconstructions were compared in different frequencies and distances. Qualitative analysis was performed by two radiologists using a four-point Likert-scale. All measured results from both quantitative and qualitative analysis were then compared between coils and clips. Results: Metal artifact index (MAI) and the intensity of coil-artifacts were significantly lower in SEMAR than in standard CTA in close vicinity to and more distally from the coil-package (*p* < 0.001, each). MAI and the intensity of clip-artifacts were significantly lower in close vicinity (*p* = 0.036; *p* < 0.001, respectively) and more distally from the clip (*p* = 0.007; *p* < 0.001, respectively). In patients with coils, SEMAR was significantly superior in all qualitative categories to standard images (*p* < 0.001), whereas in patients with clips, only artifacts were significantly less (*p* < 0.05) for SEMAR. Conclusion: SEMAR significantly reduces metal artifacts in UHR-CT-angiography images with intracranial implants and improves image quality and diagnostic confidence. SEMAR effects were strongest in patients with coils, whereas the effects were minor in patients with titanium-clips due to the absent of or minimal artifacts.

## 1. Introduction

Intracranial aneurysms are a common cerebrovascular disorder with a prevalence of around 2% [1]. Two primary options for treatment of ruptured and unruptured intracranial aneurysms are endovascular coiling and neurosurgical clipping as they prevent subarachnoid hemorrhage (SAH) and re-SAH and therefore fatal consequences [2,3]. Follow-up imaging is essential for evaluation of treatment effects, for monitoring postoperative complications, to decide whether retreatment is required, for long-term monitoring of the results, and to rule out de novo aneurysms. For these purposes, digital subtraction angiography (DSA) remains the gold standard although being invasive and potentially leading to complications [4]. Whereas magnetic resonance imaging (MRI) has been described to be well suited for follow-up imaging of aneurysms treated by coil embolization [5] it suffers from several limitations such as patient movement, metal implants, or foreign bodies incompatible with the magnetic field as well as the large artifact area surrounding the metal implant [6]. Computed tomography angiography (CTA) on the other hand is a non-invasive and extremely fast cross-sectional imaging technique, less cost intensive than MRI while offering higher spatial resolution and being available 24/7 in most hospitals. However, many metal implants causing artifacts in CTA images frequently appear as dark and bright streaks throughout the images. This may result in reduced image quality, limited assessability of relevant anatomic structures adjacent to the metal implant and therefore limited diagnostic accuracy [7].

To eliminate this shortcoming, various metal artifact reduction (MAR) algorithms and image reconstruction techniques have been developed. One of the frequently used MAR algorithms to reduce metal artifacts in CT images is the single-energy metal artifact reduction (SEMAR) algorithm [8,9,10]. 

Not long ago, an ultra-high-resolution CT (UHR-CT) scanner has been introduced in clinical practice. It generates very high quality images with a slice thickness of up to 0.25 mm, hereby allowing a detailed non-invasive cranial-imaging while improving diagnostic confidence [11,12]. 

Whereas several studies have verified the positive effects of SEMAR on CT and CTA images obtained with a conventional CT-scanner in patients with intracranial coils or clips [8,9,10,13], the efficacy of the SEMAR algorithm on UHR-CTA images with metallic artifacts from coils or clips has not been confirmed yet. As we hypothesize that SEMAR is also effective in UHR-CTA images, we aimed at retrospectively evaluating the effects of the SEMAR algorithm on CTA image quality in patients with intracranial coils or clips obtained using an UHR-CT scanner.

## 2. Materials and Methods

### 2.1. Study Design

Between November 2020 and June 2022, adult patients (*n* = 71) with intracranial aneurysms treated by coil embolization or clipping who underwent UHR-CTA in our Department of Neuroradiology were eligible for this retrospective study. Exclusion criteria were: movement artifacts, very weak contrast, the presence of coils and clips in the same vessel, and slice or the lack of SEMAR reconstructed images.

### 2.2. Ultra-High-Resolution Computed Tomography Angiography (UHR-CT)

Images were acquired using an ultra-high-resolution CT (UHR-CT) scanner (Aquilion Precision, Canon Medical Systems, Otawara, Japan) using the super high-resolution scanning mode with the following parameters: tube voltage, 120 kV; gantry rotation time, 0.35 s; beam detector collimation, 0.25 mm × 160 rows; focal spot size 0.4 × 0.5 mm; detailed pitch 0.569; field of view, 180 mm; reconstruction matrix, 1024 × 1024 pixels; slice thickness 0.25 mm. Tube current was determined individually by auto exposure control (AEC). Intravenous bolus injection of 65 mL prewarmed, nonionic contrast medium (Iopromid, Ultravist 370, Bayer Healthcare, Leverkusen, Germany) (iodine concentration of 370 mg/mL) with a high-pressure double syringe system for advanced clinical CT imaging procedures (Accutron CT-D, Medtron, Saarbrücken, Germany) at a flow rate of 5 mL/s was applied via the cubital vein through a peripheral venous catheter (18 gauge), followed by a 60 mL of 0.9% isotonic saline solution at the same flow rate. A region of interest (ROI) was placed in the aortic arch to determine the acquisition starting time. Using an automatic trigger acquisition and enhancement threshold selection the acquisition was started after a peak time attenuation threshold of 180 Hounsfield Units (HU) was reached within the ROI.

### 2.3. Post-Processing and Image Reconstruction (SEMAR)

CTA images were reconstructed according to our institution’s protocol with a slice thickness of 0.25 mm generating two sets of images: one set was reconstructed using the adaptive iterative three-dimensional dose reduction instrument (AIDR 3D standard, Canon Medical Systems Corporation, Otawara, Japan) with an FC-41 soft tissue kernel adapted for ultra-high resolution. The other set was reconstructed using the AIDR 3D standard plus the SEMAR algorithm. SEMAR is an algorithm that can be used to minimize metal artifacts in both single-energy and dual-energy CT images [8,9,10]. It identifies and segments the distorted data projection resulting from metallic implants and then modifies these data by replacing them with approximations of the corrected values using repetitive forward and backward projections and linear interpolation [8,9]. 

### 2.4. Quantitative Evaluation

Metal artifacts were evaluated quantitatively by two methods and all measured results were then compared between images with coil-artifacts and those with clip-artifacts.

**Figure 1:**
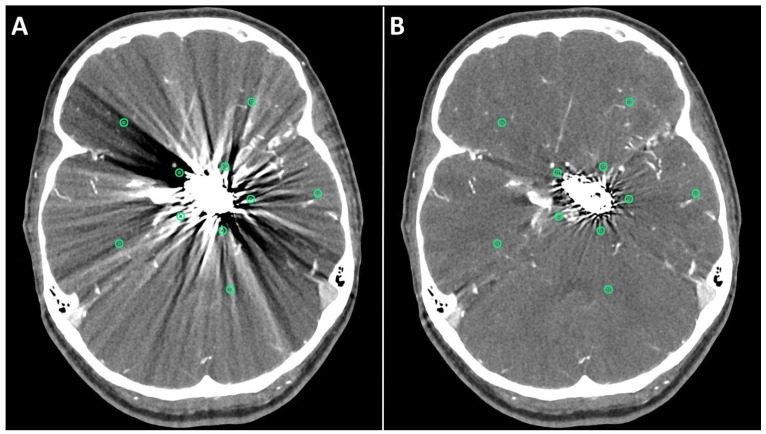
Positioning of ROIs (Region of Interest) for quantitative image analyses on standard (**A**) and SEMAR (**B**) CT-angiography images in axial plane (0.25 mm slice thickness) in direct proximity and more distally to the metal implant. In this case, follow up CT-angiography of a 56-year-old female who underwent coil-embolization of a left sided posterior communicating artery aneurysm.

The first method comprised drawing five circular regions of interest (ROIs) with a radius of approximately (10 mm each) in both standard and SEMAR CTA images around the coil/clip mass in close vicinity to the foreign body and five other ROIs more distally from the foreign body in a similar manner to previous reports [8,9,13], while focusing on the artifacts and avoiding the metallic mass, air, and bones (Figure 1). The ROI settings were constant between standard and SEMAR images. The mean density and standard deviation (SD) of each five of the five ROIs were measured in HU (Hounsfield–Units). The average image noise within the ROIs was set as the standard deviation and was considered as an index for the metal artifacts. 

For the second method, an in-house custom-built MATLAB tool (R2017b, The MathWorks, Inc., Natick, MA, USA) was used to analyze the frequencies and intensities (amplitudes) of the metal artifacts in a similar manner to previous reports [14,15]. A rough region of interest (ROI) was drawn around the coil/clip mass and its artifacts in both standard and SEMAR CTA images. The main location of the implant was determined based on the maximum HU of the ROI. In distances from 1 mm to 15 mm from the border of the implant the radial course of the signal intensity (SI) was derived, and frequencies and intensities of the artifact pattern were calculated by Fourier transformation. The frequencies were divided in 49 bins, which hold the number of artifact beams per course around the implant. The intensity of metal artifact was then compared in all the frequencies between standard and SEMAR CTA images. The difference of the metal artifacts intensity (intensity in standard images minus intensity in SEMAR images) of each patient was also measured and the median of the patients-cohort of each bin and each distance (from 1 mm to 15 mm) was calculated. The measured frequencies (bins) were divided into four different representative fields (bins 2–4: low-frequency field; bins 10–12: middle-frequency field; bins 20–22: high-frequency field; bins 40–42: very high-frequency field). The mean of each of the four representative frequency fields was then calculated and analyzed for four different distances (1, 5, 10, and 15 mm).

Furthermore, contrast-noise-ratio (CNR) and signal-noise-ratio (SNR) were measured and compared in both standard and SEMAR images. SNR and CNR were measured by drawing two circular regions of interests (ROI) with a radius of approximately (5 mm) in both standard and SEMAR images in a slice with no visible artifacts to avoid metallic artifact influence. One ROI was placed in an artery without including the vessel wall or any atherosclerotic plaques and the other ROI was placed in the brain parenchyma next to the artery while avoiding the inclusion of blood vessels. The ROI size and location were identical between standard and SEMAR images. SNR and CNR were then calculated similarly to previous reports [11,16] as follows:

$$\mathrm{SNR}=\frac{{\bar{\mathrm{HU}}}_{\mathrm{intravascular}}}{{\mathrm{SD}}_{\mathrm{parenchyma}}}$$

$$\mathrm{CNR}=\frac{{\bar{\mathrm{HU}}}_{\mathrm{intravascular}}-{\bar{\mathrm{HU}}}_{\mathrm{parenchyma}}}{{\mathrm{SD}}_{\mathrm{parenchyma}}}$$

### 2.5. Qualitative Evaluation

All images were subjectively evaluated by two board-certified radiologists (S.A. and M.A.) with many years of experience in neuroradiology after a detailed instruction. The two radiologists evaluated the following categories using a four-point Likert scale (4 being best, see Table 1) in both standard and SEMAR CTA images: overall image quality, diagnostic confidence, delineation of arteries, evaluation of the status of treated aneurysm, and severity of metal artifacts. Furthermore, they checked for the presence of any newly generated artifacts after SEMAR-reconstruction. The radiologists were free to adjust the window level and width settings for the evaluation.

### 2.6. Statistical Analysis

IBM SPSS Statistics 27.0 software package (IBM Corp., Armonk, NY, USA) was used to perform the statistical analysis. The Wilcoxon signed-rank test was used to assess the differences and calculate the significance in both, quantitative and qualitative analysis between standard and SEMAR CTA images. A *p*-value of <0.05 was considered statistically significant. In the qualitative analysis, we estimated the interrater agreement among both readers using the Cohen’s weighted kappa (κ) statistics, and we interpreted the results according to the κ values suggested by Cohen: 0.00–0.20, no to poor agreement; 0.21–0.40, fair agreement; 0.41–0.60, moderate agreement; 0.61–0.80; substantial agreement; 0.81–1.00, excellent agreement.

## 3. Results

### 3.1. Patients

A total of 71 patients were eligible for this study. Fifty-four patients (40 females; 14 males; mean age = 61.2 ± 11.9) matching the inclusion criteria were included in this study (a total of 35 patients with coils and 19 with clips). Seventeen patients were excluded due to movement artifacts, weak contrast, the presence of coils and clips in the same vessel, and slice or the lack of SEMAR reconstructed images. The used clip material was titanium in all cases (except in 2 cases). The used coil material was platinum. The treated vessels were as follows: [Internal carotid artery (ACI): 12; Middle cerebral artery (MCA): 13; Anterior communicating artery (Acom): 14; Basilar artery: 5; Posterior communicating artery (Pcom): 6; Anterior cerebral artery (ACA): 2; Posterior inferior cerebellar artery (PICA): 2]. 

### 3.2. Quantitative Evaluation

**Figure 2:**
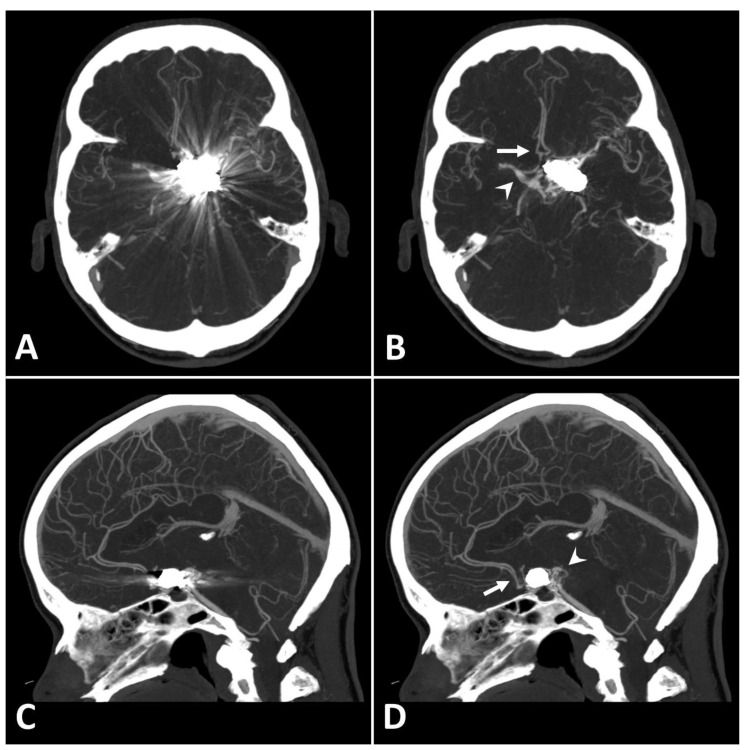
Exemplary follow up UHR-CT-angiography in axial (**A**,**B**) and sagittal (**C**,**D**) plane without (**A**,**C**) and with SEMAR (**B**,**D**) of a 56-year-old female after intracranial coil-embolization of a left sided posterior communicating artery aneurysm. All images were reconstructed as MIP (Maximum Intensity Projection) with 12.5 mm slice thickness. SEMAR significantly improves image quality, even in vessels directly adjacent to the coil package like the anterior communicating artery (white arrow in (**B**)), the contralateral terminal carotid artery (arrowhead in (**B**)), the anterior cerebral artery (white arrow in (**D**)), and the posterior communicating artery (arrowhead in (**D**)).

**Figure 3:**
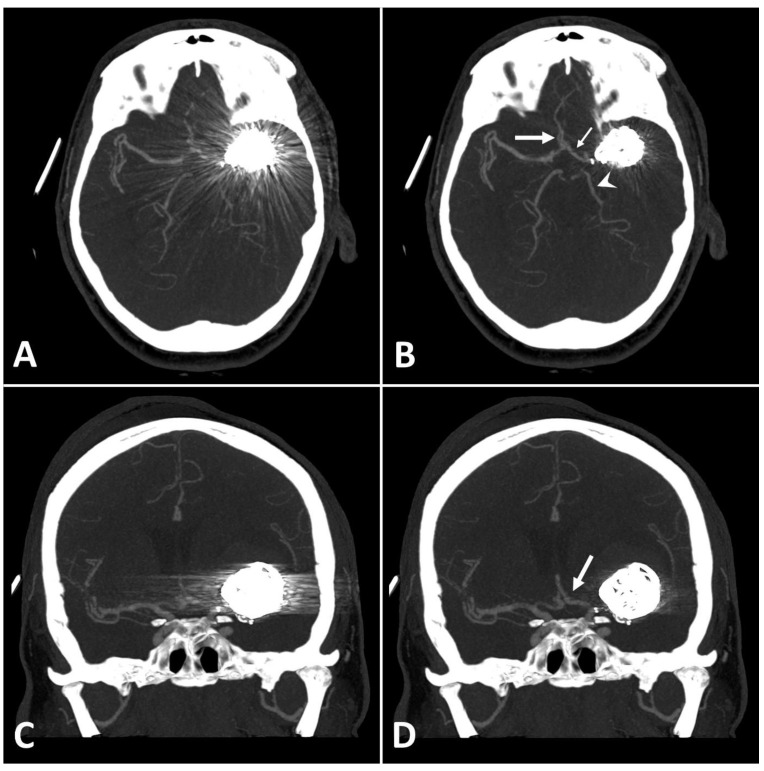
Exemplary follow up UHR-CT-angiography in axial and coronal plane without (**A**,**C**) and with SEMAR (**B**,**D**) of a 48-year-old female after intracranial coil-embolization of a left sided middle cerebral artery aneurysm, demonstrate the efficacy of SEMAR in visualizing vessels adjacent to the coil package like the ipsilateral middle cerebral artery (thick arrow in (**B**)), the anterior cerebral artery (thin arrow in (**B**)), the ipsilateral posterior cerebral artery (arrowhead in (**B**)), and the middle cerebral artery (thick arrow in (**D**)). All images were reconstructed as MIP (Maximum Intensity Projection) with 12.5 mm slice thickness.

Metal artifact index (MAI) (which was considered to be the mean image noise) was significantly reduced in SEMAR images compared to standard images in close vicinity to (70.9 ± 22.1 vs. 331.8 ± 157.5, respectively) and more distant from the coil package (24.7 ± 6.7 vs. 52.4 ± 20.3, respectively; *p* < 0.001 each). Figure 2 and Figure 3 demonstrate significantly improved image quality and visualization of arteries after applying the SEMAR algorithm on images with coil-artifacts. 

**Figure 4:**
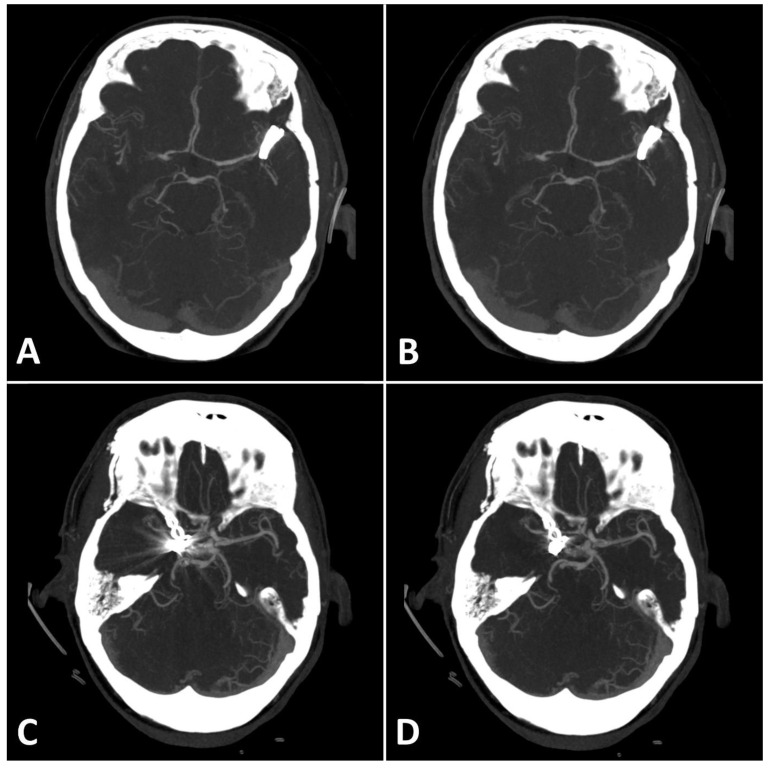
(**A**,**B**): Exemplary follow up UHR-CT-angiography in axial plane without (**A**) and with SEMAR (**B**) of a 74-year-old female with an intracranial clip in the left middle cerebral artery. (**C**,**D**): Exemplary follow up UHR-CT-angiography in axial plane without (**C**) and with SEMAR (**D**) of a 63-year-old female with an intracranial clip in the right internal carotid artery. All images were reconstructed as MIP (Maximum Intensity Projection) with 12.5 mm slice thickness and demonstrate the effects of SEMAR on images with clip-artifacts.

MAI in images with clips was significantly lower in close vicinity (SEMAR: 26.5 ± 55 vs. standard: 113.7 ± 95.4; *p* = 0.036) and more distally from the clip (SEMAR: 23.6 ± 6.5 vs. standard: 29.3 ± 16.8; *p* = 0.007). Figure 4 demonstrates the effects of SEMAR on images with clip-artifacts.

**Figure 5:**
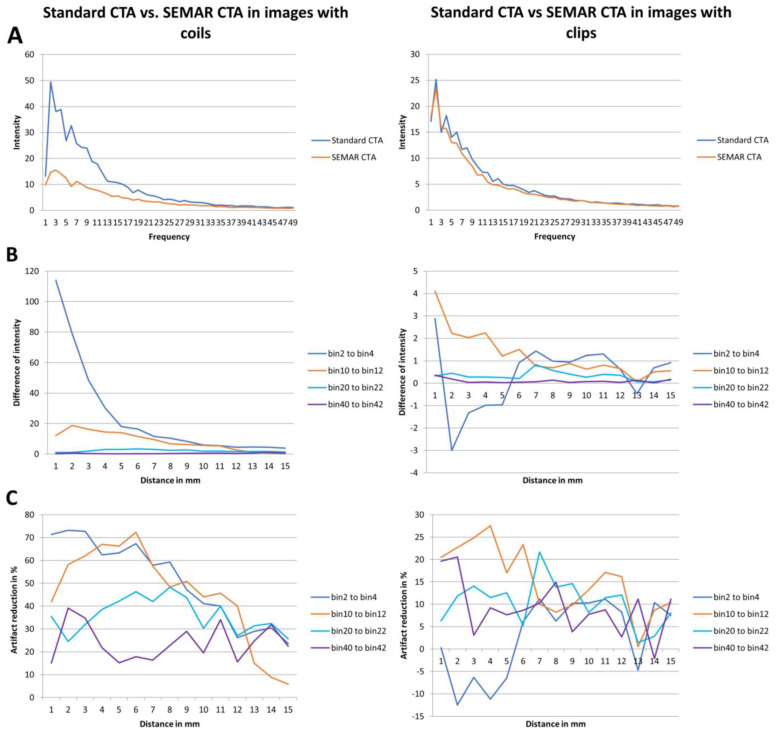
Results of frequency and intensity analyses in images with coils/clips. (**A**) Intensity of the metal artifacts of standard and SEMAR CTA images in different frequencies (bin 1 to 49). (**B**) Differences of the metal artifacts intensity in the four representative frequency fields in different distances (1 to 15 mm). (**C**) Artifact reduction of % in the four representative frequency fields in different distances (1 to 15 mm).

Analyzing the intensities (amplitudes) of metal artifacts from coils showed significantly lower artifact rates in SEMAR CTA images in all measured 49 frequencies and 15 distances compared to standard CTA images (mean = 4.4 ± 4.1 vs. mean = 9.7 ± 11.6, respectively; *p* < 0.001, both) (Figure 5A,B). The difference in intensities between SEMAR and standard images with coil-artifacts was strongest in the low frequency-field (bins 2–4) compared to middle-, high- and very high-frequency fields (bins 10–12, bins 20–22, bins 40–42, respectively) (Table 2; Figure 5B). Metal artifact reduction decreased with increasing frequency and distance to the coil package (Table 2; Figure 5C).

The intensity (amplitudes) of metal artifacts from clips was significantly less in SEMAR images compared to standard images in all measured frequencies and distances (mean = 4.5 ± 5.2 vs. mean = 5 ± 5.6, respectively; *p* < 0.001, both) (Figure 5A). The difference in intensities between SEMAR and standard images with clip-artifacts was strongest in the middle frequency-field (bins 10–12) compared to low-, high- and very high-frequency fields (bins 2–4, bins 20–22, bins 40–42, respectively) (Table 2; Figure 5B). In the low frequency field (bins 2–4) in direct vicinity to the clip, SEMAR aggravated the intensity of metal artifacts (Figure 5B,C). Whereas all images with coils benefitted from applying the SEMAR algorithm, only 58% of the images with clips benefitted from the algorithm (Figure 5C). 

Furthermore, no significant differences were observed for CNR and SNR between SEMAR and standard CTA images in images with coil packages (CNR: 28.9 ± 15.2 vs. 29.1 ± 15.2; SNR: 32.7 ± 16 vs. 33 ± 16, respectively; *p* > 0.1, each) as well as in images with clips (CNR: 32.3 ± 11.6 vs. 31.5 ± 10.9; SNR: 36.7 ± 13.1 vs. 35.8 ± 12.4, respectively; *p* > 0.1, each).

### 3.3. Qualitative Evaluation

**Figure 6:**
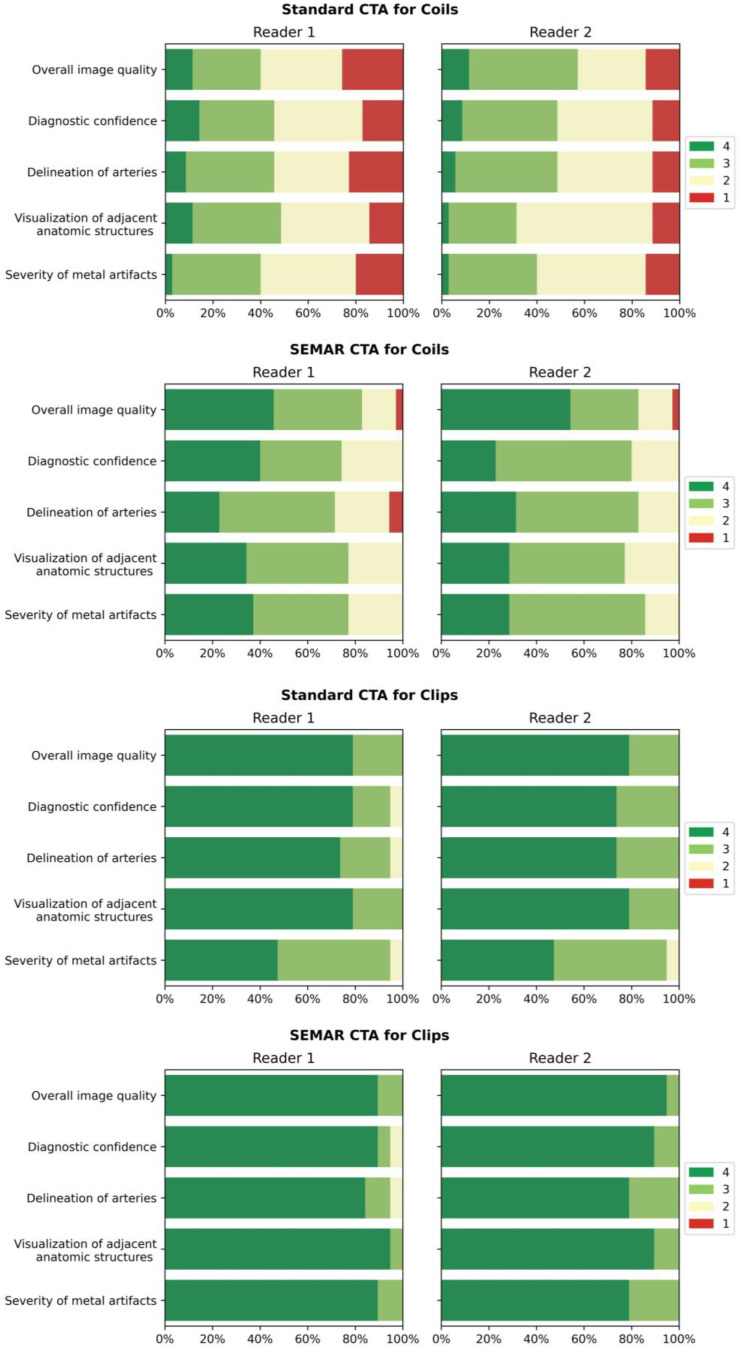
Bar-charts of Likert scale values (4 being best) rated by the readers for both standard and SEMAR CTA images with coil/clip artifacts demonstrate the distribution for the following categories: overall image quality, diagnostic confidence, delineation of arteries, visualization of adjacent anatomic structures, and severity of metal artifacts.

The results used in the following section are summarized for both readers. Table 3 shows a detailed overview of the results from the qualitative image analysis using a four-point Likert scale. Furthermore, Figure 6 visualize the results from the qualitative image analysis. 

Images with coil-artifacts, which were reconstructed with the SEMAR algorithm, showed significantly fewer metal artifacts (median in SEMAR = 3 vs. median in standard = 2; *p* < 0.001, for both readers) and had a significantly better overall image quality (median in SEMAR = 3.5 vs. median in standard = 2.5; *p* < 0.001, for both readers). Diagnostic confidence, delineation of arteries and the visualization of adjacent anatomic structures were also significantly improved in SEMAR images with coil-artifacts compared to standard images (median in SEMAR = 3 vs. median in standard = 2; *p* < 0.001, for both readers). None of the reviewers noticed any newly-developed artifacts after SEMAR-reconstruction during the analysis of both image reconstructions of patients with coils.

Images with clips showed fewer metal artifacts after reconstruction with SEMAR (median in SEMAR = 4 vs. median in standard = 3; *p* < 0.05, for both readers). However, the difference was minor compared to images with coils. There were no significant differences evaluated in all other categories in images with clip-artifacts between both reconstructions (median in SEMAR = 4 vs. median in standard = 4; *p* > 0.05, for both readers) (Table 3; Figure 6). Some minor newly-induced artifacts were noticed in some cases of patients with clips.

According to the κ values used in this study, the interrater agreement among the two readers was fair to substantial (κ = 0.516–0.835) for the categories overall image quality and diagnostic confidence and it was fair (κ = 0.508–0.594) for the other categories in both SEMAR and standard images with coil-packages. In images with clips, the interrater agreement was moderate to substantial (κ = 0.612–0.869) for all categories evaluated in both SEMAR and standard images. 

## 4. Discussion

It is essential to do long-term follow-up CTA examinations on patients with implanted intracranial coils or clips in order to detect potential risks, such as aneurysmal recanalization, re-hemorrhage, or infarction due to coil impaction as early as possible and to prevent serious complications [17,18,19,20,21]. However, metal artifacts from coils or clips complicate the evaluation of the treated vessel and implanted metal [8,9,14,22,23]. As previously reported in recent publications, MAR algorithms and techniques in general are excellent tools for reducing metal artifacts and improving image quality in many clinically relevant imaging modalities, such as MRI, FD-CT, and CB-CT [8,9,10,13,22,24,25,26,27]. Several studies have proven the positive effects of the SEMAR algorithm on CT and CTA imaging of different parts of the body with different implants obtained with standard CT scanners, as well as flat-panel scanners [8,9,10,13,24]. To the best of our knowledge, this is the first study to evaluate the effects of the SEMAR algorithm on image quality and diagnostic confidence in CTA images of the UHR-CT-scanner of patients with intracranial coils or clips.

Similarly to the conclusions of previous studies [8,13,28], we confirm that SEMAR significantly reduces metal artifacts and image noise and therefore improves image quality and diagnostic confidence in images with coil-artifacts. On contrary to other MAR algorithms such as the IMAR, which has been proven that it may compromise the contrast of vessels adjacent to the coil-package in CTA images [29], the SEMAR algorithm reduced coil-artifacts without compromising areas with no metal artifacts or developing new ones. Therefore, SEMAR made it possible for radiologists to make a sufficient diagnosis and evaluation of the treated vessel. In general, SEMAR was more effective in suppressing metal artifacts from coils than those from clips. This is due to the different ways involved in producing the metal artifacts, such as beam hardening, photon starvation, scattering, and many others [7,8,9,10,13]. In contrast to the commonly used titanium clips with low atomic number, which mostly cause minor beam-hardening effects [7,30], a major part of metal artifacts from platinum coils with higher atomic number is caused by photon starvation, which can be reduced very successfully using the SEMAR algorithm [7,8,9,10,13,31]. In accordance with the findings of prior research [14], it was noticed that the metal artifact reduction in images with coils was strongest in the low frequency-field in the area close to the coil package and it decreased with increasing frequency and distance from the metallic coil. This is due to metal artifacts from coils being mostly in low-frequencies, less in middle- and high-frequencies. With increasing frequency, fewer metal artifacts and more image noise were measured and therefore less artifact reduction was measured. This was represented in the very high-frequency field, where only very few left-over metal artifacts were measured. On the other Hand, SEMAR was effective in most cases of images with clips and the reduction of metal artifacts was strongest in the middle frequency-field, yet in some cases, it did not improve the artifacts. This difference observed in the clip group could be due to the shape or number of the used clips, the clip-gantry angle or the location of the treated aneurysm [32]. Therefore, further studies are necessary to investigate this issue.

SEMAR is an adequate tool to ensure proper evaluation and sufficient diagnosis of the treated vessel and surrounding structures. The advantages of the SEMAR algorithm are that it can be applied retrospectively to any CT image at any time without increasing the radiation dose nor image noise. In most cases, it takes a few minutes to reconstruct an image with the SEMAR algorithm in our institute. Since platinum coils cause only mild artifacts in MRI and sever artifacts in CT-imaging, MRI would be more suitable for follow up examinations of aneurysms treated by coil embolization [5,33,34] while taking the limitations of MRI into consideration. However, SEMAR makes it also possible to do a follow up CTA examination on patients with coils and allows a sufficient diagnosis to be made. On the other hand, titanium clips produce severe artifacts in MRI and remarkably less artifacts in CT-imaging [6,35]. This makes CT imaging the method of choice for follow-up CTA examinations on patients with intracranial clips while taking the radiation of CTA into consideration.

This study has some limitations. First, we were limited by the retrospective design of our study and by the small sample size. Second, we did not compare the SEMAR algorithm with the digital subtraction angiography (DSA) to confirm that images reconstructed with SEMAR reflect the true status of the treated vessel. Third, we did not compare the effects of SEMAR with other metal artifact reduction algorithms. Therefore, further comparative studies should be performed to address the mentioned issues.

## 5. Conclusions

SEMAR significantly reduces metal artifacts in UHR-CT-angiography images with intracranial implants and improves image quality and diagnostic confidence. SEMAR effects were strongest in patients with coils, whereas the effects were minor in patients with titanium-clips due to the absent of or minimal artifacts.

## Figures and Tables

**Table 1:** Four-point Likert scale and the evaluated categories of the qualitative evaluation.

	Overall Image Quality	Diagnostic Confidence	Delineation of Arteries	Visualization of Adjacent Anatomic Structures	Severity of Metal Artifacts
**4**	excellent	exact diagnosis possible	perfect vessel delineation	excellent	minimal artifacts
**3**	good	sufficient for diagnosis	good vessel delineation	good	mild artifacts
**2**	average	limited diagnostic confidence	average vessel delineation	average	strong artifacts
**1**	poor	insufficient for diagnosis	poor vessel delineation	poor	extensive artifacts

**Table 2:** Results of the intensity- and frequency-analysis of the quantitative image evaluation: differences of intensity in the four representative frequency fields in four different distances and the mean intensity in all frequency fields and all distances. Data shows mean ± standard deviation.

**Coils**				
**Difference of Intensity in:**	**1 mm**	**5 mm**	**10 mm**	**15 mm**
**low-frequency field (bin 2 to 4)**	114 ± 23.6	18 ± 7.9	5.9 ± 3.1	3.8 ± 3.3
**middle-frequency field (bin 10 to 12)**	12.1 ± 9.3	14 ± 3.5	5.6 ± 2	0.5 ± 0.7
**high-frequency field (bin 20 to 22)**	1 ± 0.3	3 ± 1.9	1.8 ± 1.4	1.3 ± 0.4
**very high-frequency field (bin 40 to 42)**	0.1 ± 0.2	0.1 ± 0.1	0.4 ± 0.4	0.5 ± 0.4
**Clips**				
**Difference of Intensity in:**	**1 mm**	**5 mm**	**10 mm**	**15 mm**
**low-frequency field (bin 2 to 4)**	2.9 ± 13.9	−1 ± 1.8	1.2 ± 1.7	0.9 ± 1.2
**middle-frequency field (bin 10 to 12)**	4.1 ± 3.5	1.2 ± 1	0.6 ± 0.3	0.6 ± 0.3
**high-frequency field (bin 20 to 22)**	0.3 ± 0.8	0.3 ± 0.2	0.3 ± 0.2	0.2 ± 0.2
**very high-frequency field (bin 40 to 42)**	0.4 ± 0.6	0.1 ± 0.1	0.1 ± 0.1	0.2 ± 0.1

**Table 3:** Results of the qualitative image evaluation. Data shows frequency of the Likert scale scores in each category of the qualitative image evaluation and the calculated median and interquartile range.

	Coils							
	Reader 1				Reader 2			
Frequency (1/2/3/4)	Non-SEMAR	Median (IQR)	SEMAR	Median (IQR)	Non-SEMAR	Median (IQR)	SEMAR	Median (IQR)
**Overall image Quality**	9/12/10/4	2 (2)	1/5/13/16	3 (1)	5/10/16/4	3 (1)	1/5/10/19	4 (1)
**Diagnostic confidence**	6/13/11/5	2 (1)	0/9/12/14	3 (2)	4/14/14/3	2 (1)	0/7/20/8	3 (0)
**Delineation of arteries**	8/11/13/3	2 (1)	2/8/17/8	3 (1)	4/14/15/2	2 (1)	0/6/18/11	3 (1)
**Visualization of adjacent anatomic structures**	5/13/13/4	2 (1)	0/8/15/12	3 (1)	4/20/10/1	2 (1)	0/8/17/10	3 (1)
**Severity of metal artifacts**	7/14/13/1	2 (1)	0/8/14/13	3 (1)	5/16/13/1	2 (1)	0/5/20/10	3 (1)
	**Clips**							
	**Reader 1**				**Reader 2**			
**Overall image Quality**	0/0/4/15	4 (0)	0/0/2/17	4 (0)	0/0/4/15	4 (0)	0/0/1/18	4 (0)
**Diagnostic confidence**	0/1/3/15	4 (0)	0/1/1/17	4 (0)	0/0/5/14	4 (1)	0/0/2/17	4 (0)
**Delineation of arteries**	0/1/4/14	4 (1)	0/1/2/16	4 (0)	0/0/5/14	4 (1)	0/0/4/15	4 (0)
**Visualization of adjacent anatomic structures**	0/0/4/15	4 (0)	0/0/1/18	4 (0)	0/0/4/15	4 (0)	0/0/2/17	4 (0)
**Severity of metal artifacts**	0/1/9/9	3 (1)	0/0/2/17	4 (0)	0/1/9/9	3 (1)	0/0/4/15	4 (0)

## Data Availability

The data used in this study are available on a reasonable request from the corresponding author.

## Abbreviations

ACA	Anterior Cerebral Artery
ACI	Internal Carotid Artery
Acom	Anterior Communicating Artery
CNR	Contrast-Noise-Ration
CTA	Computed Tomography Angiography
DSA	Digital Subtraction Angiography
HU	Hounsfield-Units
MAI	Metal Artifact Index
MAR	Metal Artifact Reduction algorithm
MCA	Middle Cerebral Artery
MRI	Magnetic Resonance Imaging
Pcom	Posterior Communicating Artery
PICA	Posterior Inferior Cerebellar Artery
ROI	Region of Interest
SAH	Subarachnoid Hemorrhage
SEMAR	Single-Energy Metal Artifact Reduction algorithm
SNR	Signal-Noise-Ratio
UHR-CT	Ultra-High-Resolution Computed Tomography
UHR-CTA	Ultra-High-Resolution Computed Tomography Angiography
